# Treatment satisfaction in Chinese medicine outpatient care: a comparison of patients’ and doctors’ views

**DOI:** 10.1186/s12906-019-2729-8

**Published:** 2019-11-06

**Authors:** Yanhong Zhang, Jiqian Fang, Wei Gao, Ying Han, Runshun Zhang, Liyun He, Baoyan Liu

**Affiliations:** 1 Institute of Basic Research in Clinical Medicine, China Academy of Chinese Medical Sciences, Beijing, 100700 China; 2 School of Public Health, Sun Yat-sen University, 74 Zhongshan RoadII, Guangzhou, 510080 China; 3 Ba Li Zhuang Community Health Service Center, Yanjingxili #11, Beijing, 100025 China; 4 Institute of Acupuncture and Moxibustion, China Academy of Chinese Medical Sciences, Beijing, 100700 China; 5 Guanganmen Hospital, China Academy of Chinese Medical Sciences, Beixiange #5, Beijing, 100053 China; 6 China Academy of Chinese Medical Sciences, Beijing, 100700 China

**Keywords:** Treatment satisfaction, Patient satisfaction, Doctor satisfaction, Outcome measure

## Abstract

**Background:**

Both doctors’ and patients’ opinions are important in the process of treatment and healthcare of Chinese medicine. This study is to compare patients’ and doctors’ treatment satisfaction over the course of two visits in a Chinese medicine outpatient setting, and to explain their respective views.

**Methods:**

Patients’ chief complaints were collected prior to the outpatient encounter. The doctor was then asked (through a questionnaire) to state what complaints he or she was prioritizing during the process of diagnosing disease and making a prescription for herbal medicine or acupuncture treatment. On the next visit, both the patient and the doctor completed a questionnaire assessing satisfaction with the treatment of Chinese medicine prescribed in the first visit and administered by the patient at home. A 5-point Likert scales was used to assess the patients’ and doctors’ satisfaction with treatment. The timing of the follow-up appointment was determined by the doctor. One chief specialist, one associate chief specialist and one attending practitioner in Chinese medicine, and 60 patients having a follow-up appointment with one of the doctors, participated in the study.

**Results:**

For 11 patients, their most urgent complaint was different from what the doctor’s choose to focus on in his or her treatment. And only one patient refused to comply due to his or her dissatisfaction with the treatment focus of the doctor. Overall, 59 patients completed the satisfaction assessment, and 53 patients visited their doctors for a follow-up appointment. Patients’ total satisfaction was higher than their doctors’ (mean 3.55 vs. 3.45), and correlation of patients’ and doctors’ treatment satisfaction was moderate (r = 0.63, *P* < 0.01). Both of the patients’ and doctors’ satisfaction ratings were correlated with treatment adherence (*P* < 0.001). The predictors of their treatment satisfaction were different. Doctors’ satisfaction with treatment was a significant factor in the process of making further clinical decisions.

**Conclusion:**

Patients and doctors form their opinion about the treatment effects in different ways. When evaluating treatment satisfaction, doctor’s opinions are also an important indicator of positive or negative clinical effects and affect the subsequent decisions-making.

## Background

Satisfaction is a widely used indicator of the quality of inpatient as well as outpatient care. In studies concerning quality of care, patient satisfaction is usually taken into account, while doctors’ opinions are considered much less often. In traditional Chinese medicine, both patients’ and doctors’ perspectives are considered to be important during the process of treatment. For example, in pre-modern times, doctors of Chinese medicine primarily used two methods for evaluating effectiveness [1]. One was oral reports of the subjective observations of patients regarding their experiences and feelings; and the other was doctor’s observations and his or her evaluations of whether these signs and symptoms indicated pathological changes. Apparently, doctor satisfaction offers important explanatory and evaluative insights into the process of medical care. In addition, we found in the previous study, that, what is valuable about the treatment effect may not always be immediately apparent to the patient [2]. But a skilled doctor will monitor the patient’s health status, noting even slight changes, to learn how to discern patterns, formulate an appropriate herbal or acupuncture point prescription and assess the patient throughout the treatment process. Therefore, we suggest taking account of the doctors’ perspective on the treatment effect in addition to the patients’ views, particularly in regard to traditional Chinese medicine.

At present, most studies have analyzed treatment satisfaction from the patients’ point of view, but only a few have discussed other perspectives, such as doctors, parents, or caregivers [3–5].. These studies could potentially show that there may be important differences between patients’ and doctors’ perceptions of the treatment process. In Chinese medicine, there are also some studies which have considered doctors’ reports regarding the care they provided [6–9], and in a few studies they have explicitly examined whether patients’ and doctors’ reports about treatment effects are congruent. All of these studies were conducted in specific disease practice, and domains are diverse from each other. Symptoms are the most common focus of doctors’ reports, but western physical examination [7], tongue observation and pulse palpation [8] were also significant. There have been no previous studies to specifically discuss and compare the doctors’ and patients’ reports after treatment, for this reason, we designed this study for such a comparison. The purpose of this study was, first, to compare patients’ and doctors’ satisfaction with the session of consultation and treatment. Second, we wanted to explain agreement or disagreement by gaining insight into the factors evaluating patients’ and doctors’ satisfaction.

The selection of predictors of satisfaction was mainly based on the literature on patient satisfaction [10] and clinical experience [11]. Patients’ health status investigation and treatment effect assessment are part of the content of the patient-reported outcomes (PROs). Signs and symptoms that patients are most painful and need to improve are usually the aim of treatment for patients, which are also the main content of the curative effect evaluation. Treatment of traditional Chinese medicine is to adjust the patients’ health status whole. When the primary symptoms are improving, the patient’s other discomforts often go better. Therefore, patients’ concomitant symptoms and systemic state are also the content of curative effect evaluation. In addition, the patient’s behavior and attitude to the treatment are the embodiment of the treatment effect, such as treatment adherence, willingness to continue treatment, etc.

For the predictors of doctors’ satisfaction with the individual treatment, it is proved based on “Chinese medicine pattern differentiation”, we have used the general domains (effectiveness, safety, satisfaction) we investigated and described in the previous study [12]. Effectiveness is a doctor’s opinion or point of view on his patient’s overall health status, and mainly based on the patient’s primary symptom, accompanying symptom and overall experience to be assessed. “Safety” refers to the adverse reactions caused by dialectical medication or other factors, when patients appear new signs or symptoms it is needed to analyze the causes of this kind of performance. “Satisfaction” is the comparison between the expected effect and the actual effect, which affects the doctor’s next clinical decisions.

Based on this, a set of questionnaires which compares and distinguishes between patients’ and doctors’ satisfaction with treatment was developed to explore and analyze their respective experiences after treatment.

## Methods

### Ethical approval

The study was approved by the Medical Ethics Committee of the Guang’anmen Hospital, affiliated with China Academy of Chinese Medical Sciences. Written informed consent to participate in the study was obtained of all respondents at each interview appointment.

### Study design

We conducted a combined with qualitative and quantitative descriptive study in which a semi-structured questionnaire evaluation on patients with Chinese medicine treatment and their doctors were performed.

### Study population

This study took place at three outpatient departments: Guang’anmen Hospital affiliated to China Academy of Chinese Medical Sciences (CACMS), Acupuncture Hospital of CACMS, and Ba-li-zhuang Community Health Service Center at the Chaoyang district in Beijing. Three doctors who specialized in general internal Chinese medicine, spleen and stomach diseases, acupuncture operation were separately invited to participate in this study in 2016, and all of them gave consent. Considering the number of predictors to be used, our aim was to recruit sixty (60) patients, with twenty (20) patients per doctor. Patients having a follow-up appointment with these doctors were contacted at the follow-up appointment. Patients were eligible if they were able to speak, read, and write Chinese and were willing to provide written informed consent.

Participating patients were asked to complete a baseline questionnaire and self-evaluation prior to the encounter. During the encounter, the doctor gave his evaluation of the patient’s current status and made treatment decisions. On the next visit, both the patient and the doctor completed a short questionnaire about the treatment satisfaction. In addition, participating doctors were also requested to complete a baseline questionnaire assessing their background characteristics on a separate occasion. If the patient didn’t re-visit his doctor at the suggested time, a follow-up call was conducted to ask for the reason and collect the patient’s feedback to assess the treatment effect up to that point. But in this case, since the doctor had no way to evaluate the patient’s health status accurately, the treatment satisfaction from the doctor’s view couldn’t be assessed. Every patient survey took less than 5 minutes. Because the doctor’s survey involved differentiating patterns, it was longer, but did not exceed thirty (30) minutes.

### Measures

#### Satisfaction

“Treatment Satisfaction Questionnaire (TSQ)” was developed to assess treatment effectiveness of Chinese medicine. This questionnaire had two parts, one was completed by patients (TSQ-P) (Additional file 1), and the other was assessed by doctors (TSQ-D) (Additional file 2). Each part had two sections, one for evaluation on current status before treatment and one for after treatment.

TSQ-P consists of six (6) items measuring patients’ satisfaction, including 1) how much the treatment resolved their primary complaints, 2) improvement of other discomforts, 3) overall health status, 4) curative effect satisfaction, 5) willingness to follow the treatment, and 6) preference of this treatment. Except for the second item which was about qualitative choices, the other item answers were given on the five (5) points Likert scale. The last two items asked the patient to explain their choices in order to understand the patients’ perspective better.

Questions of TSQ-D were adapted to make them applicable as a measure of doctors’ satisfaction. For example, the question “How well did the doctor address your chief complaints?” was modified to “How well did you address the complaints of this patient?” There were totally four items of TSQ-D which were modified to measure doctors’ satisfaction. In addition, another three qualitative items were developed, including the evaluation methods of treatment effect, the understanding of new signs and symptoms, and the relationship between the next treatment decision and the current treatment effects.

#### Sample characteristics

Patients’ social demographic characteristics such as age, gender, and education were listed in the baseline. The doctors’ baseline included background characteristics such as age, gender, level of “seniority”, specialty, and experience (number of years in practice).

### Data analyses

Both quantitative analysis and qualitative analysis were used in this study. Correlations between patients’ and doctors’ treatment satisfaction were calculated to determine patient and doctor agreement. Multilevel regression modeling was used to determine predictors of patients’ and doctors’ total satisfaction. Analyses were performed for patient and doctor satisfaction using SPSS16.0.

## Results

### Demographic data

For these sixty (60) eligible patients, 73.33% were female, and the average age was fifty-two (52) years. Most of them had received the middle school or university education. Patients’ self-reported primary symptoms included multiple diseases or conditions, such as stomach pain or dyspepsia (*n* = 20), neck and shoulder pain, lumbar pain (*n* = 10), insomnia (*n* = 6), facial paralysis (*n* = 4), hearing loss or tinnitus (n = 4), post-herpetic neuralgia (*n* = 3), stroke (n = 2), chest distress or palmus (n = 2), dizziness (n = 2), prosopalgia (n = 1), common cold (n = 1), asthenia (n = 1), acne (n = 1), dropsy (n = 1),stasis (n = 1) and splenomegaly without physical symptoms or discomfort (n = 1). The patients assessed their severity prior to clinical encounter, and also their doctors gave their evaluation on the patients’ current status before treatment. The results of patients’ self-assessment were not fully consistent with the doctors’ evaluation (Kappa = 0.158). This indicated there was difference between patients’ experience and doctors’ perspective. Characteristics of participating patients are displayed in Table 1. The basic information of the three participating doctors is shown in Table 2.

**Table 1:** Sample Characteristics: Patient Demographics (*N* = 60)

	Number	Percentage/SD (Range)
Gender		
Female	44	73.33%
Male	16	26.67%
Mean age, y	52	SD12.8(24 to 82)
<25	1	1.67%
25 to 39	11	18.33%
40 to 65	40	66.67%
>65	8	13.33%
Education		
Primary school	2	3.33%
Middle school	31	51.67%
University	27	45%
The main complaints		
Stomach pain or dyspepsia	20	33.33%
Neck and shoulder pain, lumbar pain	10	16.67%
insomnia	6	10%
Facial paralysis	4	6.67%
Hearing loss or tinnitus	4	6.67%
Post-herpetic neuralgia	3	5%
Stroke	2	3.33%
Chest distress or palmus	2	3.33%
Dizziness	2	3.33%
Prosopalgia	1	1.67%
Common cold	1	1.67%
Asthenia	1	1.67%
Acne	1	1.67%
Dropsy	1	1.67%
Stasis	1	1.67%
Splenomegaly without physical symptoms or discomfort	1	1.67%
Severity-assessed by patients		
Mild	18	30%
Moderate	29	48.33%
Severe	11	18.33%
Very severe	2	3.33%
Severity-assessed by doctors		
Mild	6	10%
Moderate	26	43.33%
Severe	27	45%
Very severe	1	1.67%

**Table 2:** Sample Characteristics: Doctors (N = 3)

Work unit	Age	Gender	Education	Professional title	Specialty	Years of experience
Guang’anmen Hospital	45	Male	Doctor	Chief doctor	Spleen and stomach diseases	20
Acupuncture Hospital	41	Female	Master	Associate chief doctor	Acupuncture treatment	18
Ba Li Zhuang Community Health Service Center	33	Female	Master	Attending doctor	General internal medicine	7

### Complaints and treatment

Table 3 lists the symptoms that sixty (60) patients were most anxious to improve (chief complaints), the secondary symptoms and conditions that were also of concern, and the targeted symptoms that the doctors wanted to treat in this period of treatment. There were eleven (11) patients whose chief complaints weren’t in accord with their doctors’ concerns. Instead, these patients’ concomitant symptoms became the treatment focus. This is common in Chinese medicine because treatment is based on pattern differentiation. Of the eleven (11) patients, two patients (ID28, ID52) didn’t revisit their doctors again. By calling them we learned that, one felt better after treatment so he saw no need to further treatment, while the other expressed her dissatisfaction because she thought the doctor hadn’t made a prescription that addressed her primary complaints. In addition to these two (2) patients, another nine (9) patients continued to come to see their doctors for further treatment. Of the nine (9) patients, three (3) patients (ID44, ID52, ID53) thought their diseases were chronic, and it was necessary to continue treatment for a while to experience the curative effects. Another six (6) patients expressed their satisfaction with their current treatment.

**Table 3:** Symptoms of Patients’ Complaints and Doctors’ Treatment (N = 60)

Patient ID	Patients’ chief complaints	Secondary or additional complaints	Doctors’ treatment focus
1	Lumbar and leg pain	Poor sleep, Neck discomfort	Lumbar and leg pain
2	Hearing loss and tinnitus	Poor sleep	Hearing loss and tinnitus
3	Chest pain	–	Chest pain
4	Neck pain and lumbar pain	Knee pain	Neck pain and lumbar pain
5	Poor sleep	–	Poor sleep
6	Headache	–	Headache
7	Weakness of limbs on the right side	Waist pain and legs pain	Weakness of limbs on the right side
8	Mouth being pulled to one side	–	Mouth being pulled to one side
9	Numbness of limbs on the right side	Weakness of the right limbs	Numbness of limbs on the right side
10	Poor sleep	Headache during menstrual period, Stomach discomfort	Poor sleep
11	Deviated mouth	Neck pain, Back pain	Deviated mouth
12	Deviated mouth	–	Deviated mouth
13	Feeling blue	Headache, Constipation, Swelling in hands	Feeling blue
14	Poor sleep	Neck pain	Poor sleep
15	Poor sleep	Back pain	Poor sleep
16	Facial pain	Poor sleep, Constipation	Facial pain
17	Neck pain and back pain	–	Neck pain and back pain
18	Poor sleep	–	Poor sleep
19	Herpes Zoster on left jaw	–	Herpes Zoster on left jaw
20	Deviated mouth	–	Deviated mouth
21	Dizziness	Poor sleep, Pain and legs pain	Dizziness
22	Tinnitus	Leg pain, Lumbar soreness	Tinnitus
23*	Palpitations, Shortness of breath	Irregular menstruation, Poor sleep, Excess dreaming, Abdominal cold	Sensation of cold in the abdomen
24	Diarrhea	Dizziness, Fatigue	Diarrhea
25	Swelling on the scalp	Cough, Palpitations, Dry stool, Dry mouth	Swelling on the scalp
26	Palpitations	Neck discomfort, Knee discomfort	Paroxysmal palpitations
27	Pain and leg pain	Poor sleep, Joint pain, Constipation, Obesity	Pain and leg pain
28*	Elbow pain	Headache, Lacking in strength	Morning headache
29*	Soreness in low back	Tinnitus, Abdominal pain, Heel pain, excess dreaming	Abdominal pain
30	Palpitations	Dry eyes, Dizziness, Feeling cold	Palpitations
31	Pain and aversion to cold	Neck discomfort, Shoulder pain	Pain and aversion to cold
32*	Cold fingers and toes	Shortness of breath, Low energy,	Shortness of breath
33	Chest discomfort and palpitations	Dry stool	Chest discomfort and palpitations
34	Dizziness	Feeling cold, Heaviness of back muscles	Dizziness
35	Pain	–	Pain
36	Pain	Femur head pain	Pain
37	Stomach bloating	Vomits, Slow fecal transit	Stomach bloating
38	lumbar sprain	–	lumbar sprain
39	Adnexa uteri cysts	Stomach burning sensation	Adnexa uteri cysts
40	Back pain	Chest discomfort, Palpitations	Back pain
41*	Hiccup	Lacking in strength, Side stitches	Side stitches
42	Stomachache	Back pain, Poor sleep	Stomachache
43	Burning sensation in stomach	–	Burning sensation in stomach
44*	Fatigue	Aversion to cold, excess dreaming, Hiccups, Poor appetite, Lacking in strength	Poor appetite
45*	Stomachache	Poor appetite,	Burning sensation
		Burning sensation	
46*	Stomach burning sensation and sour regurgitation	–	Stomach bloating
47	Diarrhea	Feeling anxious	Diarrhea
48	Hypochondriac pain	Aversion to cold in low back	Hypochondriac pain
49	Stomach burning sensation and sour regurgitation	Bellyache after eating	Stomach burning sensation and sour regurgitation
50	Stomachache	No sense of defecation	Stomachache
51	Dizziness	Headache, Dry throat, Dry mouth, Swollen tongue, Low appetite, Nausea, Constipation	Dizziness
52*	General dropsy	Abdominal distension	Abdominal distension
53*	Abdominal distension	Low back pain, Bad breath	Bad breath
54	Hypochondriac pain	Red spots on the skin, Itching	Hypochondriac pain
55	High transaminase	–	Abnormal liver function
56	Abdominal distension	Stomach burning sensation	Abdominal distension
57	Enlarged spleen	Pain	Enlarged spleen
58	Hiccup	–	Hiccup
59	Bellyache	Constipation, Feeling cold, Poor sleep	Bellyache
60*	Abdominal pain	Eyestrain, Shoulder pain, Short of breath, Dizziness	Stomachache

*Patients’ chief complaints and doctors’ treatment are different

### Participants and procedure

**Fig. 1:**
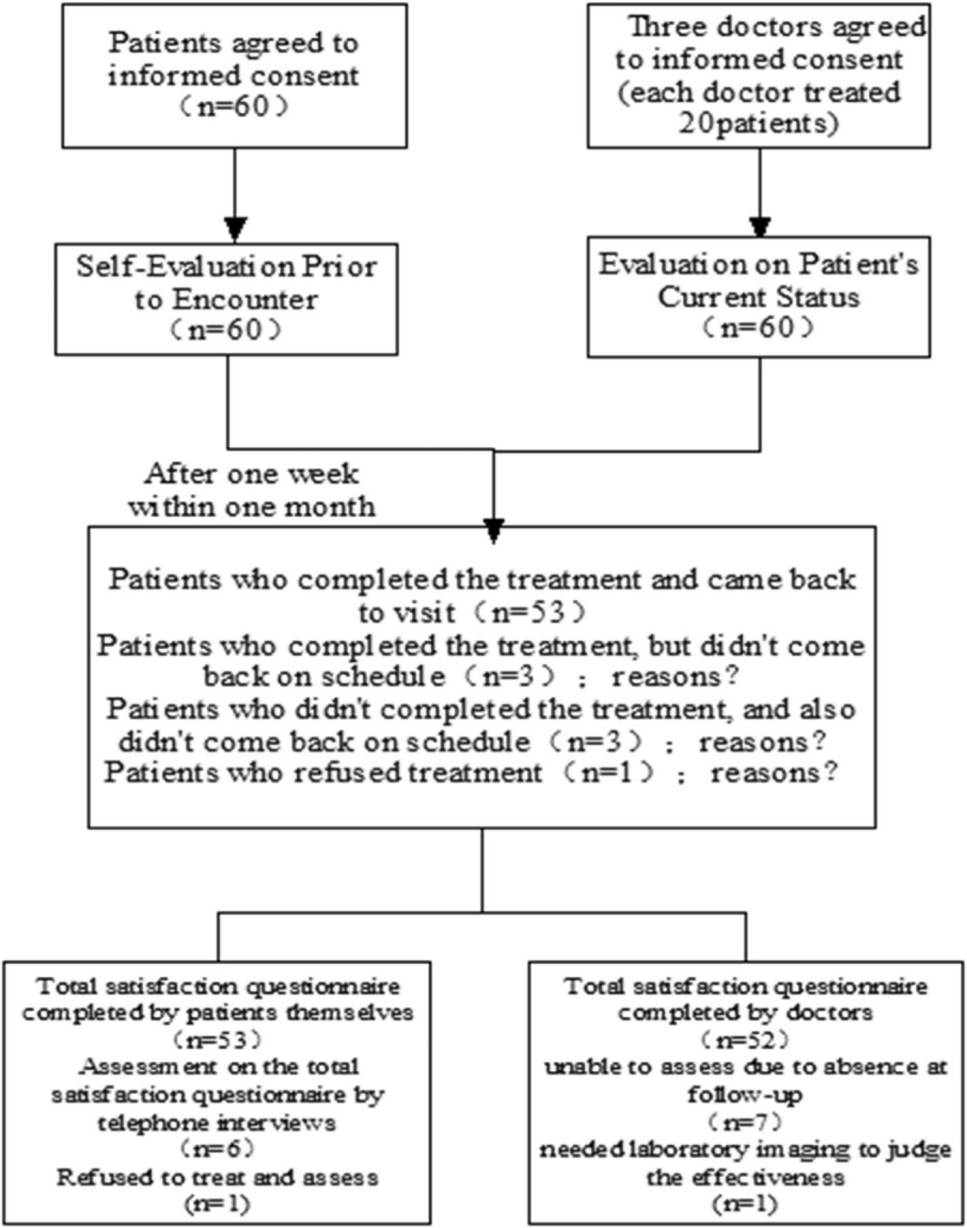
Procedure of Measures

Figure 1 shows the procedures followed in the study. First, all of the patients completed a survey in which they were asked to assess their health status; this mainly consisted of writing down what they were suffering from and most wanted to improve. After completing the survey, they were seen by the doctor who evaluated the patient in his or her usual manner and gave treatment based on Chinese medicine pattern differentiation. Treatments consisted of herbal medicine prescriptions or acupuncture. The patients were asked to return to see their doctors at a scheduled follow-up appointment. The timing of the follow-up appointment was decided by the doctor, and was usually about 7 days later, but never more than 1 month after the initial visit.

Seven (7) patients did not show up for the follow-up appointment. Telephone follow-up was used for these seven (7) patients. It was revealed that, three (3) patients complied with the treatment as directed but they didn’t come back to revisit their doctor; one stated that it was due to feeling better, and the other two reported that it was because of unsatisfactory results. Another three (3) patients hadn’t also returned for follow-up due to business obligation or other personal reasons, but they reported that they were satisfied with the current treatment. Only one (1) person refused to take the herbal prescription which the doctor gave her, and her stated reason for non-compliance was that she thought the prescription couldn’t treat her disease at all, because it was not made based on her chief complaints.

Fifty-three (53) patients received treatment and attended the scheduled follow-up appointment. The fifty-three (53) patients completed the total satisfaction questionnaire when they arrived for their appointment. Another six (6) patients who did not attend the follow-up appointment were asked to complete the questionnaire by phone. The patient who refused to take the treatment couldn’t be asked to assess her treatment satisfaction. The doctors also assessed their patients and gave them the next treatment based on pattern differentiation. Because Chinese medicine doctors must see the patients in person in order to assess the effects of the previous treatment, the other seven (7) patients weren’t assessed by their doctors. In addition, of the fifty-three (53) patients, one (1) patient sought treatment for abnormal enlargement of the spleen, and his doctor needed laboratory imaging to judge the results of treatment. In this case, the doctor could only answer the other questions, omitting the item “change of the chief complaints”. Therefore, fifty-two (52) questionnaires were fully completed both by patients and doctors.

### Patients’ and doctors’ satisfaction

**Fig. 2:**
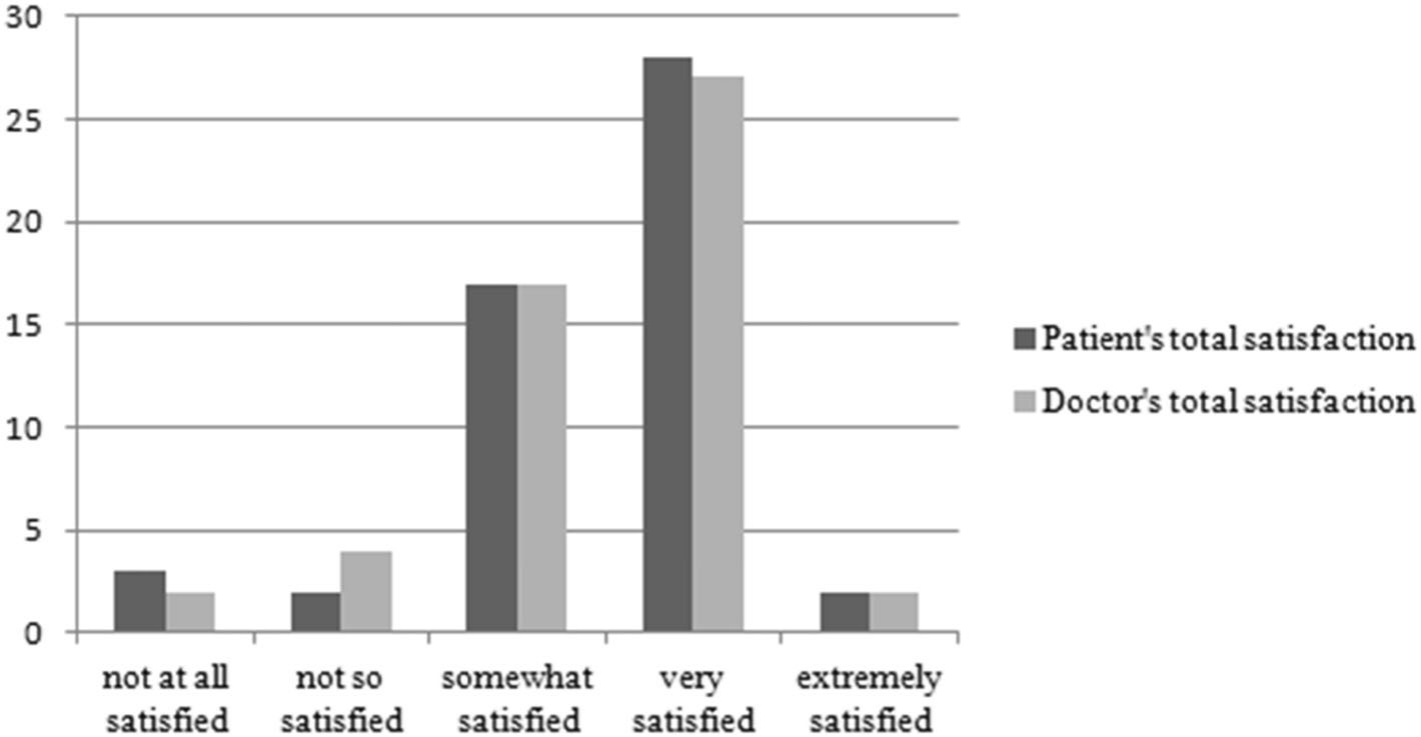
Patients’ and Doctors’ Total Satisfaction with Treatment (*N* = 52)

Patients’ and doctors’ reported treatment satisfaction is presented in Table 4 and Fig. 2. The correlation with “patients’ chief complaints” was 0.64(*P* < 0.001), indicating both doctors and patients attached importance to the patients’ main symptoms. Patients’ “overall health status” assessed by their doctors was higher than self-evaluation (mean 3.36 vs. 3.28), but correlation was also 0.64(*P* < 0.001). The correlation between doctors’ and patients’ “treatment effect satisfaction” was 0.44(*P* < 0.01), indicating a “medium-sized” association, but doctor satisfaction was substantially lower than patient satisfaction. For the “total satisfaction”, patients’ was a little higher than doctors’, and the correlation was 0.63 (P < 0.01). The correlation between each doctor (*n* = 3) and their patients as a group was not significant.

**Table 4:** Treatment Satisfaction

Satisfaction with	Patients’ Mean (SD) ^†^	Doctors’ Mean (SD) ^†^	Correlation^‡^
Patients’ chief complaints	3.27(0.98)	3.15(1.02)	0.64^***^
Overall health status	3.28(0.93)	3.36(0.79)	0.64^***^
Treatment effect satisfaction	4.10(0.85)	3.83(1.04)	0.44^**^
Total satisfaction^§^	3.55(0.87)	3.45(0.85)	0.63^**^

^***^P<0.001

^**^P<0.01

^*†*^ *5-point Likert scale. Due to missing data, n ranges from 58 to 59 (patients’) and from 52 to 53 (doctors’)*

^*‡*^ *Correlation between patients’ and doctors’ total satisfaction at visit level, accounting for the potential correlation between patients of the same doctor and the doctor*

^*§*^ *Patients’ and doctors’ average response on the 3 items*

### Factors predicting satisfaction

Predictors of patient and doctor satisfaction are shown in Table 5. Patient satisfaction was positively correlated with their treatment adherence (P < 0.001). Patients’ satisfaction was not associated with background characteristics such as their age, gender, education level, nor was it associated with their primary complaints’ severity. Patients’ preference for this kind of Chinese medicine treatment did not predict their satisfaction ratings. This model explained 53.4% of the variance in patients’ satisfaction. On the other hand, different hospitals were not associated with higher or lower patient satisfaction. We found that the severity of the condition assessed by their doctors was correlated with lower patients’ satisfaction score (*P* < 0.05), but the patients’ satisfaction was not associated with the severity assessed by themselves.

**Table 5:** Predictors of Patient and Doctor Satisfaction, Using Multilevel Multiple Regression Analysis

Final model	Patient total satisfaction (*n* = 55) Coeff(SE)	Doctor total satisfaction (*n* = 47) Coeff(SE)
Patient variables		
Age	Not selected	Not selected
Gender	Not selected	Not selected
Education	Not selected	Not selected
Severity assessed by patient	Not selected	Not selected
Adherence	0.63(0.08)^***^	0.47(0.09)^***^
Preference	Not selected	Not selected
Doctor variables		
Hospital	Not selected	−0.49(0.13)^***^
Severity assessed by doctor	−0.36(0.16)^*^	− 0.38(0.15)^*^

^*^P<0.05; ^**^P<0.01; ^***^P<0.001

Higher doctor satisfaction was associated with higher patient adherence (*P* < 0.001). Doctor satisfaction was not predicted by patients’ age, gender, education, severity assessed by patient or patient preference for the treatment. Doctors with higher hospital status may have lower treatment satisfaction ratings. In addition, the severity of the symptom or condition assessed by doctors was negatively correlated to treatment satisfaction. This model explained 37.8% of the related variables in doctors’ satisfaction.

### Treatment decision

**Fig. 3:**
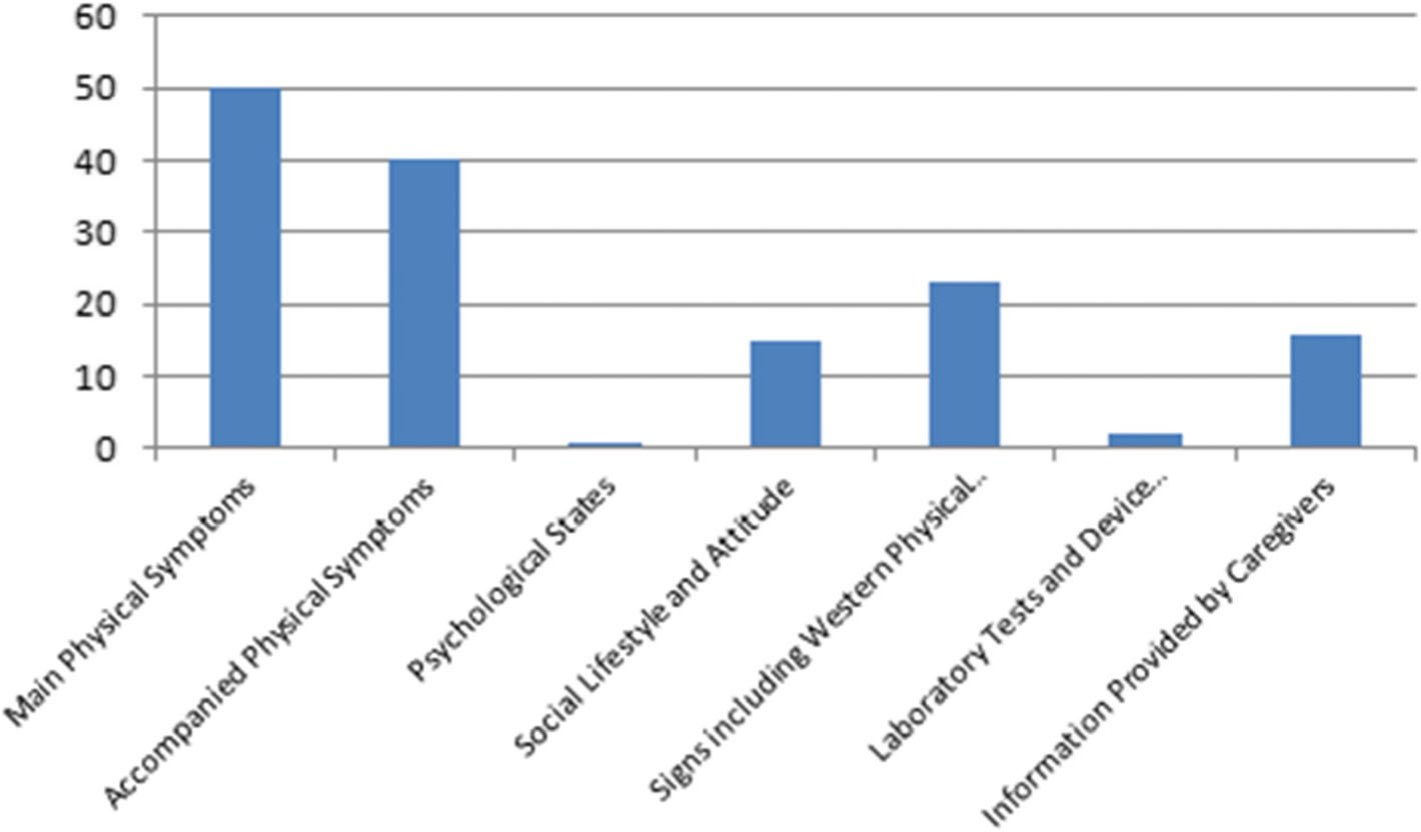
Evaluation of Treatment Satisfaction Factors

Doctor’s evaluation of treatment satisfaction was mainly based on the patient’s primary symptoms, supplemented by the other signs and findings, including western physical examination and Chinese medicine clinical manifestations. In addition, information provided by caregivers was also important when the patient has no or limited capacity to accurately report his or her experiences. However, Chinese medicine doctors (both in general, and in this particular sample) cannot make decisions based only upon laboratory tests or device measurements. For doctors, factors used to assess treatment satisfaction are shown in Fig. 3.

There were eight (8) patients who reported the appearance of some new symptoms or signs after the last treatment, such as chest pain, hiccups, throat discomfort, stomachache, abdominal distension, loose stools, etc. The doctors attributed these to the external environment (5), new diseases (2), or a positive sign related to effective treatment (1).

For the next treatment decision, these fifty-three (53) follow-up patients were treated as follows. One (1) patient was advised to terminate treatment because the patient felt well and the condition was stable. Thirty-nine (39) patients were told to continue the last treatment for a few additional courses of treatment in order to “consolidate the effectiveness” because their conditions were better, but the doctors hoped for continued improvement and to stabilize the effects. Doctors altered the treatment principles and herbal prescriptions for three (3) patients because there was no significant change in their conditions at present. Another ten (10) patients were treated for their new symptoms because their primary discomfort had been improving during the last course of treatment. To analyze the relationship between the item of “treatment effect satisfaction” and the next treatment decision, the patient’s response was divided into three groups as follows: unsatisfactory (1 = not at all satisfied, 2 = not so satisfied), general (3 = somewhat satisfied), and satisfactory (4 = very satisfied, 5 = extremely satisfied). Table 6 shows the relationship between doctors’ treatment decisions, patients’ satisfaction and doctors’ satisfaction. This clearly shows that curative effects correspond to the further treatment goals.

**Table 6:** Comparison of Patients’ and Doctors’ Treatment Effect Satisfaction with Doctors’ Follow-up Decisions

Further treatment decision		Patients’ treatment effect satisfaction			Doctors’ treatment effect satisfaction		
	*N*	Not at all or Slightly *N*(%)	Moderately *N*(%)	Very much or Extremely *N*(%)	Not at all or Slightly *N*(%)	Moderately *N*(%)	Very much or Extremely *N*(%)
Termination of treatment	1	0	0	1(100)	0	0	1(100)
Keep taking the same formula	39	1(2.6)	2(5.1)	36(92.3)	0	15(38.5)	24(61.6)
Use the formula with minor modifications	3	2(66.7)	0	1(33.3)	3(100)	0	0
Change to a new formula	10	0	1(10)	9(90)	1(10)	0	9(90)
Total	53	3(5.7)	3(5.7)	47(88.7)	4(7.6)	15(28.3)	34(64.2)

## Discussion

This study compared patients’ and doctors’ treatment satisfaction before and after an out-patient consultation consisting of pattern differentiation and prescription. Patients’ satisfaction was considerably higher than doctors’ reported levels of treatment satisfaction, which appears that doctors were more critical of their treatment than patients. The correlations between patients’ and doctors’ ratings were medium-sized, which indicates that patients and doctors actually judged the treatment effect differently. Patient satisfaction was dependent of their desire to alleviate his discomfort, whereas a doctor’s satisfaction ratings were not only dependent on the patient’s stated needs, but also took the patient’s overall health status into account. In a general practice setting, doctors integrated all factors to analyze the development and changes of patients’ health status from a holistic point of view. For doctors, all information, including patients’ feedback, the doctors’ examination, tests results or other health caregivers’ suggestions, were taken into account as factors used to evaluate treatment effect. Doctors’ emphasizes on the progression of the disease state as a whole, whereas patients’ tends to focus on subjective sensations and observations. Therefore, patients and doctors may have different perceptions of the treatment effect as a result of different expectations and demands.

On the one hand, patients and doctors agreed that the most relevant element of treatment satisfaction is patients’ adherence. But it is important to note that, in terms of adherence, patients’ own explanation of their choice indicates that having a follow-up appointment does not necessarily indicate that they think the treatment was effective or they are highly satisfied. Shorter courses of treatment, no other known better therapies, or improving overall status, were all given as possible reasons to revisit the doctor. The patients usually pay more attention to the effectiveness rather than the convenience for Chinese medicine, so expected effectiveness may likely be one of important reasons for follow-up adherence. It is certain that, patients who desired greater participation in medical decision-making were more satisfied with the visit. In other words, there is some indication that the patient-doctor acquaintance, including trust of his doctor, helps get better curative effects, even when curative effects did not appear immediately within one course of treatment.

On other hand, patients and doctors disagreed about the relative importance of the professional status of the hospital. In China, hospitals are rated as three levels. In this study, Guang’anmen Hospital is a Grade IIIA hospital, and other two are both GradeIhospitals. Particularly, doctors at high level hospital assessed treatment effect with lower satisfaction, which indicates that the doctor be more critical in higher level hospital. But for patients, the assessment results weren’t affected by the different hospitals, which means the doctor’s individual ability is more important than his external factors associated with the doctor.

As far as the severity, not only patients’ but also doctors’ treatment satisfaction couldn’t be predicated by the patients’ severity assessment. While the illness is more serious assessed by doctors, the treatment satisfaction of patients’ and doctors’ both tends to be lower. This is probably because the severity reported from patients is more subjective than that of doctors, which is consistent with our previous studies [1, 2, 8, 9]. Three doctors were selected into the study, and twenty (20) patients each doctor were reported different symptoms. The characteristics of treatment based on patterns differentiation indeed cause great challenges to evaluate the effectiveness and treatment satisfaction. It had been reported that the doctor should be considered as an important factor for individualized medicine [13]. This study also indicates that although with comparable satisfaction, there exist significant differences in various complaints, different views, and different treating principles used by the three (3) doctors.

In addition, preference is an outcome variable, and in our study, it appears to be not be associated with treatment satisfaction. Many patients expressed that, whether they would be willing to recommend their doctors to others would be based on their further experience and effectiveness. It needs to note that, patient recommends the doctor to others not the treatment, which indicate the doctor is the most important behavior practitioners in Chinese medicine. In western medicine, patients often recommend some treatments or medication to the others, while surgeons based on technical operation is more similar to Chinese medicine practitioners by mouth advertising. To some extent, the status of a doctor in his or her patient’s mind is determined by the medical professional characteristics.

It seems that the administrative status of the doctors or hospital did not affect patients’ satisfaction. In our study, there was no evidence to show patients’ behaviors are associated with doctors’ age or gender. In further investigations, we’ll increase the sample size so that these variables can be further examined, including professional title, years of working.

### Limitations

Our study has several limitations; only one section study design, thus factors affecting patients’ and their doctors’ responses cannot be studied over time. Additionally, the views as well as attitudes of the patients and doctors regarding treatment satisfaction may change over time during the treatment process, therefore, more sections longitudinal study should be performed to better explain these behaviors. Results of this survey only represent the opinions of the patients and doctors belonging to Chinese medicine hospitals, which cannot be generalized to other comprehensive medical institutions. The findings on treatment satisfaction are based on patients’ and doctors’ views who are treated in Chinese medicine hospitals, whereas the visions of patients and their doctors in combined medicine hospitals have not been described who might have different opinions. In this study, three (3) doctors with sixty (60) patients were selected to participate in the investigation. The sample size was not large. In the following studies, more larger samples will be involved and the factors influencing treatment satisfaction will be further discussed and analyzed.

## Conclusion

Treatment satisfaction often reflects the attitude of patients and doctors towards the effectiveness of the treatment and help make further treatment decisions. This study suggests that there is indeed a difference between patients’ own experience and doctors’ understanding of patients’ post-treatment results. Finding the reasons for the difference can help narrow the gap between doctors and patients to improve clinical empathy. At the same time, our findings underscore the commonly-accepted perception that in the eyes of patients seeking Chinese medicine, treatment methods are inseparable from the doctor. To some extent, doctors themselves represent the intervening measure, so patients are more concerned to which doctor to see rather than which treatment to choose. This is not surprising given the individualized nature of Chinese medicine diagnosis and treatment and the nature of the decision-making process. Previous studies also analyzed the importance of doctor satisfaction as a factor in communication between doctors and patients [14]. In traditional Chinese medicine, doctor satisfaction not only affects doctor-patient communication, but also influences diagnosis and subsequent treatment decisions. We suggest that in real world studies, effectiveness from the perspective of doctors should also be included in outcome measurement and staged treatment assessment.

## Supplementary information

**Additional file 1.** Treatment Satisfaction Questionnaire of Patient (TSQ-P).

**Additional file 2.** Treatment Satisfaction Questionnaire of Doctor (TSQ-D).

## Data Availability

Upon request to the corresponding author at dalifortune@126.com

## Abbreviations

CACMS: China Academy of Chinese Medical Sciences
PROs: Patient-reported outcomes
TSQ: Treatment Satisfaction Questionnaire
TSQ-D: Treatment Satisfaction Questionnaire by Doctors
TSQ-P: Treatment Satisfaction Questionnaire by Patients
