# Supplementary material for: Treatment satisfaction in Chinese medicine outpatient care: a comparison of patients’ and doctors’ views
Source: BMC Complement Altern Med. 2019 Nov 6;19:300. doi: 10.1186/s12906-019-2729-8 (PMC6836653; doi:10.1186/s12906-019-2729-8)
Supplement: Supplementary file 1 — Additional file 1. Treatment Satisfaction Questionnaire of Patient (TSQ-P). [file 12906_2019_2729_MOESM1_ESM.docx]

**Additional file 1**

**Treatment Satisfaction Questionnaire of Patient (TSQ-P)**

**(English version)**

| **Self-Evaluation Prior to Clinical Encounter** | | | |
| --- | --- | --- | --- |
| Describe your current health status: | | | |
| 1 | | The most urgent symptom that you would like to improve, is____________  (write only one symptom) | |
| 2 | | For the discomfort mentioned above, the severity is_______  □mild □moderate □severe □very severe | |
| 3 | | If you have other health problems, please write them here.  ________________________ | |
| **Treatment Assessment by Patient** | | |  |
| Looking back at the treatment you have been given by the doctor: | | |  |
| 1 | How well did the doctor address your chief complaints?  □not at all well □not so well □somewhat well □very well □extremely well | |  |
| 2 | Do you feel any improvement in other areas of your body?  □Yes, I feel better in the areas of ________________________.  □No, I don’t feel any change.  □I don’t feel any other discomfort other than my main complaint. | |  |
| 3 | How much better do you feel about your health status?  □not at all better □not so better □somewhat better □much better □extremely better | |  |
| 4 | How satisfied are you with the last curative effect?  □not at all satisfied □not so satisfied □somewhat satisfied  □very satisfied □extremely satisfied | |  |
| 5 | How willing are you to continue with the treatment given by your doctor last time?  □not at all willing □not so willing □somewhat willing □very willing □extremely willing  Please explain your choice: ________________________. | |  |
| 6 | How willing are you to recommend this treatment to others?  □not at all willing □not so willing □somewhat willing □very willing □extremely willing  Please explain your choice: ________________________. | |  |

**患者治疗满意度调查问卷**

**(中文版)**

| **就诊时的病情状态（患者填写）** | | | |
| --- | --- | --- | --- |
|  | | | |
| 1 | | 您此次来看病，最迫切、最需要改善的是：____________(只填写一个症状) | |
| 2 | | 您上面提到的不适，请评估一下严重程度：□轻度 □中度 □较重 □严重 | |
| 3 | | 如果您还有其他方面的身体不适，请全部写下来。________________________ | |
| **治疗效果评估（患者填写）** | | |  |
|  | | |  |
| 1 | 您最迫切、最需要改善的症状，经过治疗以后缓解了吗？  □根本没缓解 □很少缓解 □缓解（一般）□多数缓解 □完全缓解 | |  |
| 2 | 您觉得您身体其他方面的不适有改善吗？  □有改善 □没有改善 □无其他不适  如果您选择“有改善”，请告诉我们在哪些方面有改善？____________________ | |  |
| 3 | 您感觉您的身体状况好多了吗？  □根本没好 □很少好 □好（一般）□多数好 □完全好了 | |  |
| 4 | 总体来讲，您对上次的治疗效果满意吗？  □很不满意 □不满意 □既非满意也非不满意 □满意 □很满意 | |  |
| 5 | 您愿意继续使用上次医生给予的治疗方法吗？  □根本不愿意 □很少愿意 □愿意（一般）□比较愿意 □极愿意  请对您的选择进行说明：________________________. | |  |
| 6 | 您愿意将这种疗法推荐给其他人吗？  □根本不愿意 □很少愿意 □愿意（一般）□比较愿意 □极愿意  请对您的选择进行说明：________________________. | |  |

| **Appendix 2**  **Treatment Satisfaction Questionnaire of Doctor (TSQ-D)**  **(English version)**   \| **Evaluation of Patient’s Current Status by Doctor** \| \| \| --- \| --- \| \| Describing the patient’s current health status: \| \| \| 1 \| What do you think is the patient’s most urgent symptom that you would like to improve, ____________(write only one symptom) \| \| 2 \| For the symptom you mentioned above, please assess the severity:_______  □mild □moderate □severe □very severe \| \| 3 \| Please write any other symptoms of the patient here, including the main and the secondary symptoms  Main symptoms:________________________;  Secondary symptoms: ________________________. \| | | |
| --- | --- | --- | --- | --- | --- | --- | --- | --- | --- | --- | --- | --- |
| **Treatment Assessment by Doctor** | |  |
| Looking back at the treatment you have given to the patient: | |  |
| 1 | How well did you address the chief complaints of this patient?  □not at all well □not so well □somewhat well □very well □extremely well |  |
| 2 | Do you believe that any improvements have occured in other areas of this patient’s body?  □Yes, I believe some improvement has occurred in the areas of________________________.  □No, I don’t believe any change has occurred.  □This patient doesn’t feel any other discomfort but the main needs. |  |
| 3 | How much better do you feel this patient’s health status is?  □not at all better □not so better □somewhat better □much better □extremely better |  |
| 4 | You assess the treatment effect of this patient based on:  □Main physical symptoms  □Accompanied physical symptoms  □Psychological states  □Social lifestyle and attitude  □Signs including western physical examination and Chinese medicine clinical manifestations  □Laboratory tests and device measurements  □Information provided by caregivers |  |
| 5 | How satisfied are you with the effects of the last treatment?  □not at all satisfied □not so satisfied □somewhat satisfied □very satisfied □extremely satisfied  If you think the actual effect is affected by some other factors, please write it here: _______________________. |  |
| 6 | If some new symptoms or signs appeared after the last treatment, please write it /them here: ________________________.  You think the new symptoms or signs emerging are due to:  □due to the typical course of disease, the treatment is likely to be effective.  □inaccurate pattern differentiation.  □constitution of the patient.  □external environment.  □misuse of herbs, such as inappropriate compatibility, inferior quality and improper decocting procedures, etc.  □Chinese medicine combined with Western medicine.(drug interactions)  □other drugs being used concomitantly to treat other diseases.  □other newly diagnosed diseases. |  |
| 7 | Please give further treatment recommendations for this patient:  □To terminate treatment because the patient feels well and the condition is stable.  □To use the last treatment for a few additional days or weeks to consolidate the effectiveness because the patient’s condition is better.  □To use the last treatment for a few additional sessions although there is no significant change in the patient’s condition at present.  □To modify the prescription based on the previous principle because there is no significant change in the patient’s condition at present.  □To change the strategy and treatment because there is no significant change in the patient’s condition at present.  □To adapt thinking and treatment to the new circumstances because the patient’s condition is better.  Please explain your choice: ________________________. |  |

| **医生治疗满意度调查问卷**  **(中文版)**   \| **就诊时的病情状态（医生填写）** \| \| \| --- \| --- \| \|  \| \| \| 1 \| 您此次来看病，最迫切、最需要改善的是：____________(只填写一个症状) \| \| 2 \| 您上面提到的症状，请评估一下严重程度：□轻度 □中度 □较重 □严重 \| \| 3 \| 如果患者还有其他的主症及伴随症状，请如实填写。  主症：________________________ 伴随症状：________________________ \| | | |
| --- | --- | --- | --- | --- | --- | --- | --- | --- | --- | --- | --- | --- |
| **治疗效果评估（医生填写）** | | |
| 1 | 您认为，患者最迫切、最需要改善的症状缓解了吗？  □根本没缓解 □很少缓解 □缓解（一般）□多数缓解 □完全缓解 |  |
| 2 | 患者的其他主症和/或伴随症状有改善吗？  □有改善 □没有改善 □无其他不适  如果您选择“有改善”，请告诉我们在哪些方面有改善？_____________________ |  |
| 3 | 您认为患者的身体状况好多了吗？  □根本没好 □很少好 □好（一般）□多数好 □完全好了 |  |
| 4 | 对于该患者的疗效，您的判断依据有哪些？  □主症 □伴随症状 □患者的心理 □患者的社会功能 □医生的体检 □实验室指标 □照顾者或家属提供的信息 |  |
| 5 | 您认为，患者的实际疗效符合您的治疗预期吗？  □根本不符合 □很少符合 □符合（一般）□多数符合 □完全符合  如果您认为患者的实际疗效受到了其他因素的影响，请写下来。________________ |  |
| 6 | 如果患者治疗以后，出现了新的症状或体征，请逐一列出，并给出原因。  新出现的症状或体征：____________________  □与病机转归吻合，治疗有效 □失治或误治 □患者体质 □外界刺激  □中药使用不当，如药材质量、配伍、煎服法等 □中西药合并用药互相影响  □患者服用其他药物引起 □患者出现了新的疾病 |  |
| 7 | 请给出您对患者的进一步处理措施，并说明原因。  □病情改善，终止治疗 □病情改善，续用前方巩固  □病情未见明显变化，续用前方 □病情未见明显变化，治法不变，调整处方  □病情未见明显变化，调整辨证思路，改变治法  □病情改善，调整治疗方向，改变治法  请对您的选择进行说明：____________________ |  |
